# Digital ischemia after lidocaine with epinephrine injection in a patient with primary Raynaud’s phenomena

**DOI:** 10.1080/23320885.2022.2117702

**Published:** 2022-09-04

**Authors:** Colin T. McNamara, Mark Greyson

**Affiliations:** Division of Plastic and Reconstructive Surgery, University of Colorado Anschutz Medical Center, Aurora, CO, USA

**Keywords:** Epinephrine, digital block, Raynaud’s, epidermolysis, necrosis, finger

## Abstract

Lidocaine with epinephrine is ubiquitous in hand procedures. Although existing literature supports the overall safety of this, significant physiologic changes accompanying epinephrine can disproportionately affect vascularly compromised patients, such as in Raynaud’s phenomenon. The literature is reviewed and a case presented regarding the dangers of epinephrine injection in this population.

## Introduction

Injection of lidocaine for analgesia has become common-place in surgery for the hand. The use of epinephrine concomitantly has been of continued debate since its inception due to the theoretic concern of vasoconstriction leading to necrosis. Raynaud’s phenomenon is a vasospastic condition affecting approximately 3–5% of the population [1] and divided into primary Raynaud’s, which is idiopathic, and secondary, which has an underlying cause. Both have decreased arterial inflow in specific areas related to thermoregulation and heightened sympathetic response [2]. Either of these may exacerbate the effect of epinephrine in the finger and leading to significant injury.

Here we present a case highlighting this potential and review the existing literature on the use of epinephrine for digital blocks, particularly in this population.

## Case

A 72 year-old, non-smoking female with a history of hyperlipidemia, hypertension, heterozygous for Factor V Leiden, deep venous thrombosis not on anticoagulation, and a transient ischemic attack 10 years prior to exam presented for triggering of her bilateral ring fingers. No specific information regarding a history of Raynaud’s phenomena was elicited prior to presentation for surgery. She was treated with corticosteroid injections however seven months later she noted recurrence and was diagnosed with Green’s grade 2 trigger finger of her right long finger. She desired surgical intervention in wide-awake clinic. Analgesia was obtained *via* 3 mL of 1% lidocaine with 1:100,000 epinephrine injected directly over the A1 pulley. Five mL of 0.25% Marcaine without epinephrine was injected at the conclusion of the case in the same location. Dressings were confirmed to be wrapped loosely at the end of the procedure.

**Figure 1.**
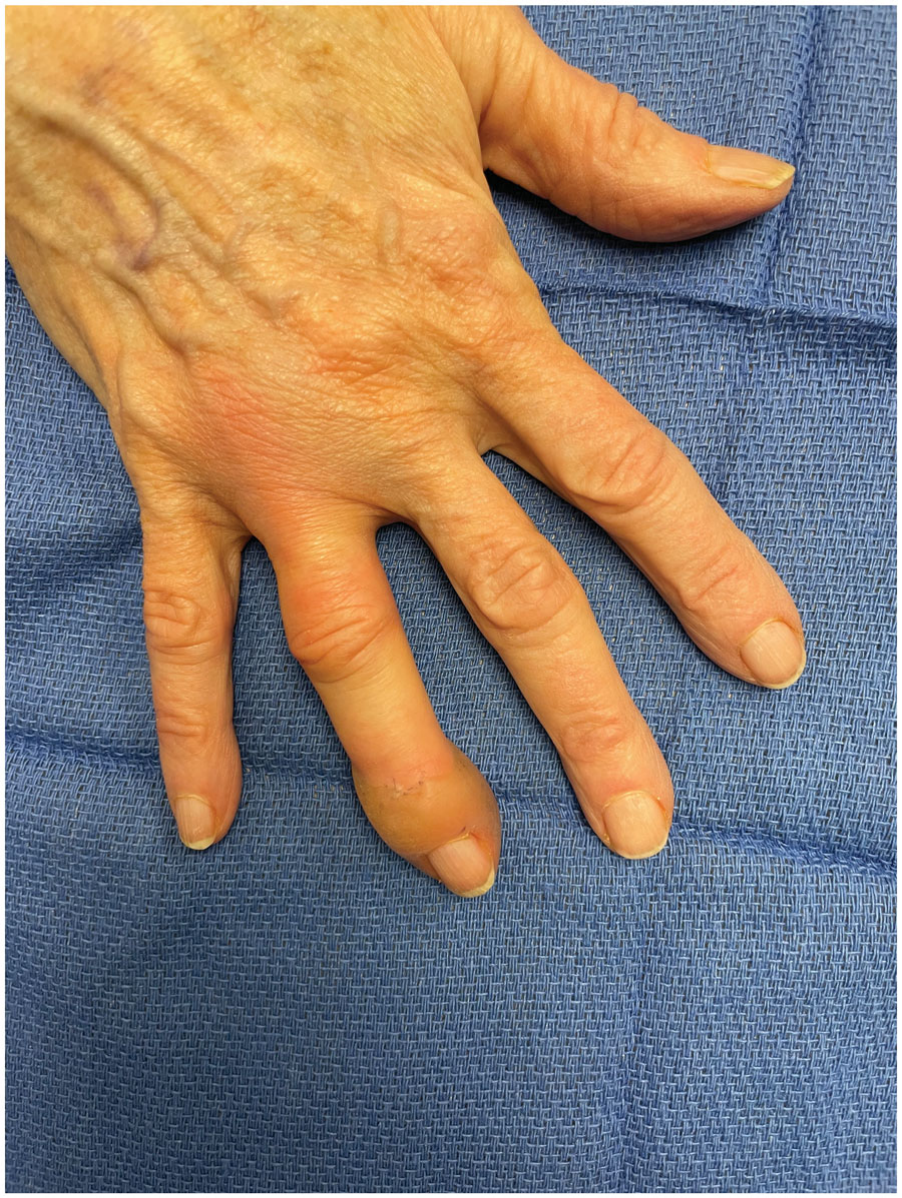
Bullous blisters on ring finger POD 1 from trigger finger release.

**Figure 2.**
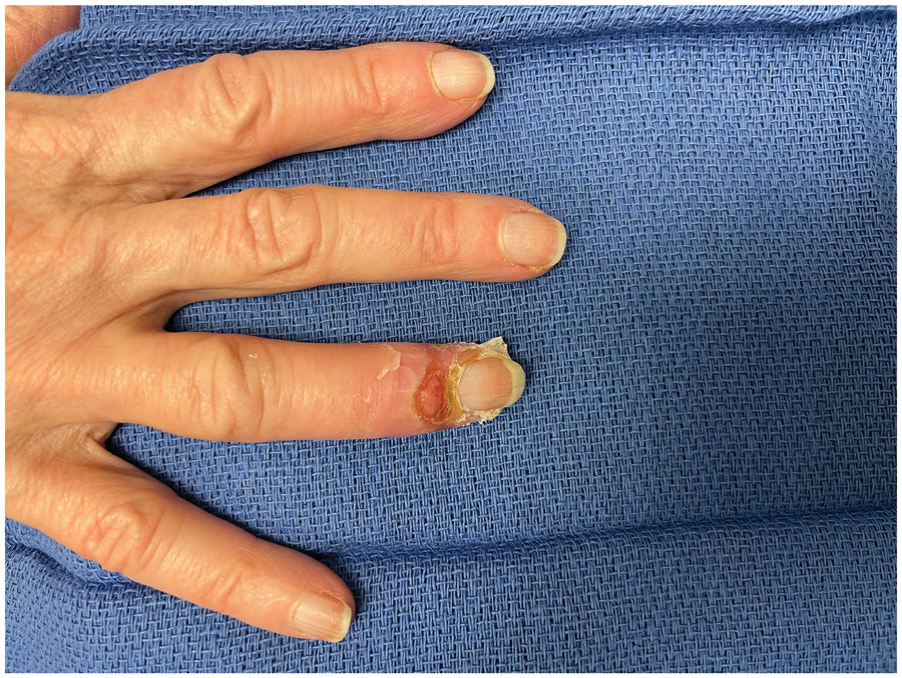
POD 7 following fenestration of bullae and evolution of superficial wounds.

**Figure 3.**
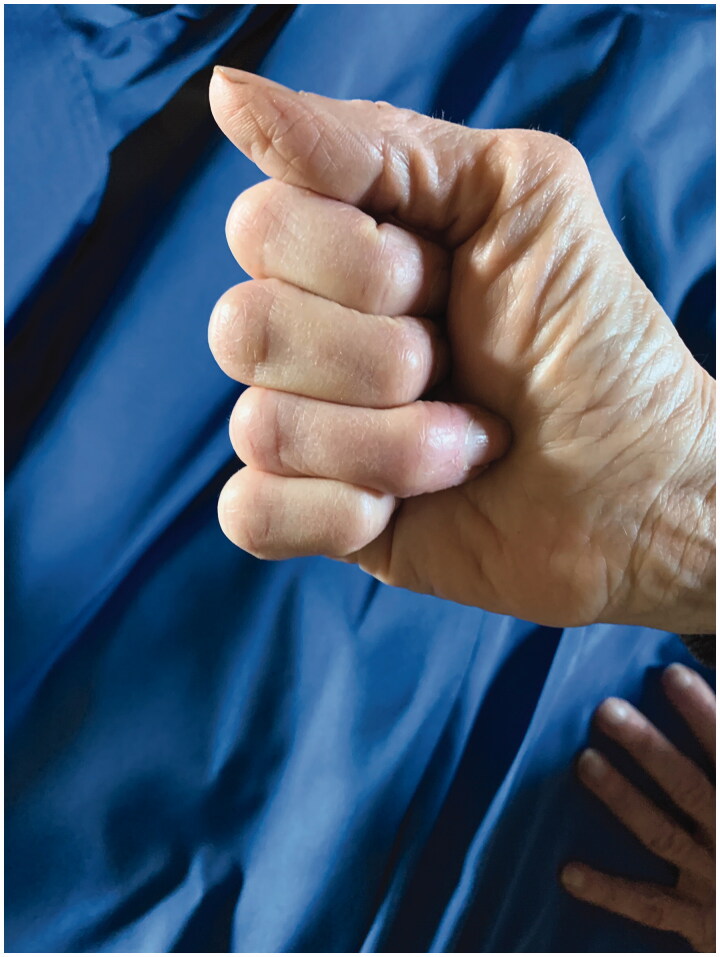
Healed wounds with persistent stiffness.

Patient called in that evening with swelling and stating it had been white for the rest of the day. She was advised to apply heat and noted temporary improvement; however by the next morning she had developed a large bulla at the tip of the finger. She was promptly assessed in the clinic (Figure 1). The finger was warm and well perfused and the decision was made not to inject phentolamine. The bullae were fenestrated and daily dressing changes were performed. After additional questioning she reported vasospastic changes to her fingers after cold exposure consistent with primary Raynaud’s phenomena. Over the next 2 weeks her epidermolysis evolved and sloughed (Figure 2). By 4 weeks the wounds were healed however she had persistent stiffness and difficulty with range of motion at her distal interphalangeal joint (Figure 3).

## Discussion

Henrich Braun added epinephrine to cocaine in 1903 in order to create a ‘chemical tourniquet’ and prolong the anesthesia effect [3]. However he had persistent concerns regarding the possibility of the vasoconstriction leading to gangrene [4,5]. Initial case reports seemed to validate this concern but on retrospective review, of the 48 cases found before 1948, many used procaine or cocaine (which can cause digital infarction) versus lidocaine, only 21 used epinephrine and typically in unknown concentrations, and many had concurrent risks including using hot soaks, infection and tight tourniquets [4,6]. Since 1948 and the creation of commercially available combination lidocaine and epinephrine products, there have been very few reported complications and numerous retro- and prospective studies that emphasize the safety of epinephrine use in the digits of the hand and foot [6]. Even in the cases of high dose (1:1000) epi-pen accidental injection to the digits, many of whom were not treated with phentolamine, none developed permanent sequaele [7–9].

A number of physiologic studies have also been performed that indicate the epinephrine creates only transient vasoconstriction that returns to baseline within several hours and typically within 1 h [10–13]. When examined closely however, these studies still present findings that may be concerning when applied to at-risk populations. Several articles have found that after epinephrine injection there are changes in distal oxygen saturation [13,14].

Moog et al. utilized an injection of 5–7 mL of 1% lidocaine with 1:200,000 epinephrine and continuously monitored oxygenation. They showed significant reductions of >30% in venous saturation occurred in 7 of their 17 patients and critical levels (<10% sO_2_) in 4/17. All levels returned to normal within 30 min but each decline had an average length of 132.5 s for a total average length of 463 s per patient. The longest patient had a total critical ischemia time of 684 s [14]. Alitinyazar et al. also showed in 4 out of their 24 patients that though vasoconstriction resolved in 60–90 min, 4 patients had no measurable flow by Doppler at 10 min [12].

The majority of these prospective, retrospective and physiologic studies exclude patients with vascular disease or compromise, which includes Raynaud’s phenomenon. Given Raynaud’s phenomena patients have been shown to have increased response to alpha agonism [15] and an increased density of alpha receptor levels [16], the alpha-agnosim of epinephrine may result in longer critical ischemia and secondary injury.

Several case reports have been published that emphasize this potential. Four cases of ischemic injury are reported in the literature; in all of these, as with ours, the diagnosis of primary Raynaud’s phenomenon was discovered after the injury [17–20]. Two other cases in the literature demonstrate digital ischemia with a similar symptom sequence after epinephrine injection although they do not explicitly state there was a history of Raynaud’s or that there was specific questioning regarding previous signs or symptoms [21,22].

All of the cases demonstrate that the ischemic change happens remarkably quickly, with duskiness proceeding to bullae or necrosis within hours. This supports the hypothesis that although these ischemic injuries may be brief, given their increased susceptibility it does not take significant injury to push them into necrotic territory. Recovery in these cases was mixed; some young, healthy patients required amputation and older, arteriosclerotic patients were able to heal. Half of the patients, including our patient, were older with hypertension, dyslipidemia and some level of diagnosed arteriosclerosis. This may represent a confounder or potentially a second hit hypothesis in which risk is additive for vascular occlusion. In addition, our patient was heterozygous for Factor V which has been found to be a risk for large vein thrombosis [23] but remains more unclear in the setting of microvascular circulation or repair [24].

Anecdotally in this series patients improved with vasodilatory therapy [18]. Only one patient was given phentolamine but was noted to have marked improvement afterward [19]. Phentolamine, a competitive alpha-receptor antagonist, has been shown to reduce the duration of epinephrine-induced vasoconstriction in fingers [25] and its use is encouraged in cases of extended vasoconstriction secondary to epinephrine.

Overall despite the evidence that epinephrine is safe there are clear physiologic changes that accompany injection, even in the vascularly normal patients studied. This transient but potentially significant ischemic time combined with the pathophysiology of Raynaud’s may increase the responsiveness to epinephrine and lead to injury. We therefore encourage excellent history and physical exam for signs or symptoms of Raynaud’s phenomenon in all patients undergoing digital blocks. We strongly caution against the use of epinephrine in digital blocks for these patients and the use of non-epinephrine analgesia such as wide-awake anesthesia with a tourniquet if any concern exists.
